# Bioinformatics resources for deciphering the biogenesis and action pathways of plant small RNAs

**DOI:** 10.1186/s12284-017-0177-y

**Published:** 2017-08-07

**Authors:** Dongliang Yu, Xiaoxia Ma, Ziwei Zuo, Weishan Shao, Huizhong Wang, Yijun Meng

**Affiliations:** 1 College of Life and Environmental Sciences, Hangzhou Normal University, Hangzhou, 310036 People’s Republic of China; 2 Zhejiang Provincial Key Laboratory for Genetic Improvement and Quality Control of Medicinal Plants, Hangzhou Normal University, Hangzhou, 310036 People’s Republic of China

**Keywords:** Database, Software, Next-generation sequencing (NGS), Biogenesis, Action, Plant small RNA

## Abstract

The next-generation sequencing (NGS) technology has revolutionized our previous understanding of the plant genomes, relying on its innate advantages, such as high throughput and deep sequencing depth. In addition to the protein-coding gene loci, massive transcription signals have been detected within intergenic or intragenic regions. Most of these signals belong to non-coding ones, considering their weak protein-coding potential. Generally, these transcripts could be divided into long non-coding RNAs and small non-coding RNAs (sRNAs) based on their sequence length. In addition to the well-known microRNAs (miRNAs), many plant endogenous sRNAs were collectively referred to as small interfering RNAs. However, an increasing number of unclassified sRNA species are being discovered by NGS. The high heterogeneity of these novel sRNAs greatly hampered the mechanistic studies, especially on the clear description of their biogenesis and action pathways. Fortunately, public databases, bioinformatics softwares and NGS datasets are increasingly available for plant sRNA research. Here, by summarizing these valuable resources, we proposed a general workflow to decipher the RDR (RNA-dependent RNA polymerase)- and DCL (Dicer-like)-dependent biogenesis pathways, and the Argonaute-mediated action modes (such as target cleavages and chromatin modifications) for specific sRNA species in plants. Taken together, we hope that by summarizing a list of the public resources, this work will facilitate the plant biologists to perform classification and functional characterization of the interesting sRNA species.

## Review

### Introduction

Ten years after the accomplishment of the first plant genome project (Mozo et al., 1999), the advent of the next-generation sequencing (NGS) technology has uncovered an unprecedentedly intricate scene of genome-wide transcription in plants (Varshney et al., 2009; Kelly and Leitch, 2011; Jain, 2012). In addition to the already annotated protein-coding genes, the fact is emerging that millions of the non-coding RNAs (ncRNAs) are transcribed from the intergenic or the intragenic regions (Jiang, 2015; Wendel et al., 2016). These non-coding transcripts could be roughly classified into the long non-coding RNAs (lncRNAs; > 200 nt) (Chekanova, 2015) and the small non-coding RNAs (sRNAs; < 200 nt) (Chen, 2009). Owing to the relatively short read length of NGS, the sRNAs were easier to be cloned at the beginning of the plant ncRNA research. Expectedly, the explosive sRNA world immediately became a hot research topic for the plant biologists. Notably, some of these small transcription “noises”, which were once regarded as the degraded remnants, have been demonstrated to be generated through specific pathways and play essential roles in plant development (Chen, 2009).

**Fig. 1:**
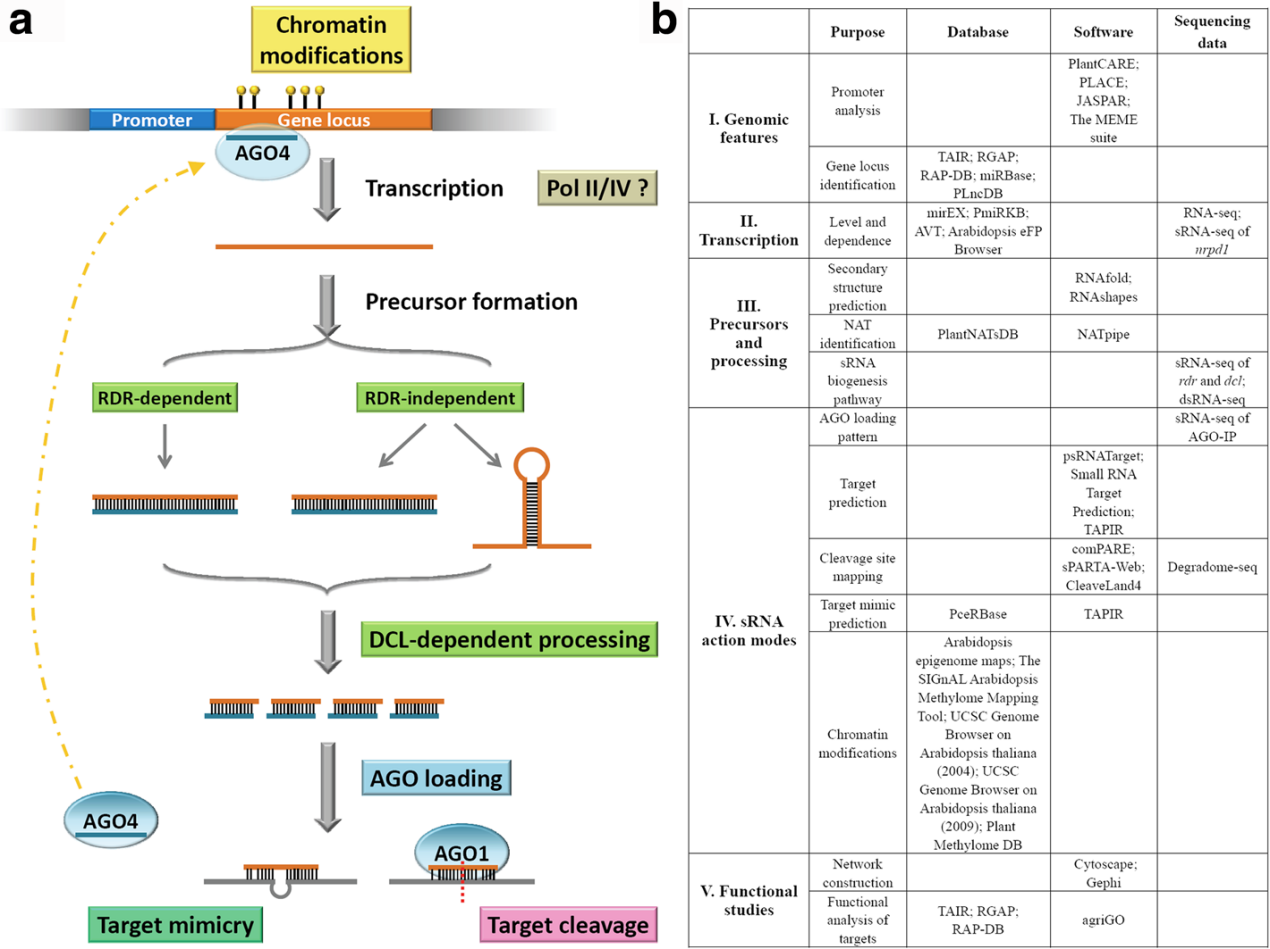
A general workflow for public bioinformatics resource-based investigation of small RNA (sRNA) biogenesis and action pathways in plants. **a** Presents a brief framework of the biogenesis and action pathways of plant sRNAs. AGO: Argonaute; Pol: RNA polymerase; RDR: RNA-dependent RNA polymerase; DCL: Dicer-like; sRNA: small RNA. **b** The analysis is divided into five sections according to the step-by-step instructions in the main text, including “genomic features”, “transcription”, “precursors and processing”, “sRNA action modes” and “functional studies”

One of the well studied sRNA species is microRNA (miRNA) (Jones-Rhoades et al., 2006; Voinnet, 2009). In plants, the transcription of most miRNA genes is driven by RNA polymerase II (Pol II), resulting in the production of the 5′ capped and 3′ poly(A) (polyadenylation)-tailed transcripts called primary microRNAs (pri-miRNAs). Relying on the highly complementary base pairing, stable hairpin-like structures could form within the specific regions of the pri-miRNAs. These local hairpin structures are the featured substrates of Dicer-like 1 (DCL1). Followed by the DCL1-mediated two-step cropping in the nucleus, the pri-miRNAs are sequentially processed into the secondary precursors named as precursor microRNAs (pre-miRNAs), and then into the miRNA/miRNA* duplexes. After exporting to the cytoplasm, the mature miRNAs are selectively loaded into specific Argonaute (AGO)-centered protein complexes. In most cases, the miRNAs will be recruited by AGO1, although some exceptional cases have been reported for “miR172—AGO10” and “miR165/166—AGO10” in Arabidopsis (*Arabidopsis thaliana*), “miR168—AGO18” in rice (*Oryza sativa*), and “miR390—AGO7” in both plants (Fang and Qi, 2016). The AGO complex is guided by the recruited miRNA to bind onto a specific target transcript containing a region highly complementary to the miRNA. There are two major action modes of miRNA-guided gene silencing in plants. One is target cleavage which is considered as the most common mode (Voinnet, 2009), and the other is translational repression which has been observed in several studies (Chen, 2004; Gandikota et al., 2007; Li et al., 2013). Another class of plant sRNAs is collectively referred to as the small interfering RNAs (siRNAs), which could be further classified into heterochromatic small interfering RNAs (hc-siRNAs), *trans*-acting small interfering RNAs (ta-siRNAs), natural antisense transcript-derived small interfering RNAs (nat-siRNAs), and phased small interfering RNAs (phasiRNAs). Specifically, hc-siRNAs are encoded within the heterochromatic loci transcribed by RNA Pol IV. The single-stranded Pol IV transcripts are converted to double-stranded precursors through the RDR2 (RNA-dependent RNA polymerase 2)-dependent pathway. Then, the precursors are processed by DCL3 for the production of the 24-nt hc-siRNAs. The hc-siRNAs are incorporated into AGO4 to perform site-specific chromatin modifications (Xie et al., 2004; Qi et al., 2005; Henderson et al., 2006). In Arabidopsis, there are four *TAS* gene loci encoding ta-siRNAs. MiRNA-mediated cleavages (miR173 for *TAS1* and *TAS2*, miR390 for *TAS3*, and miR828 for *TAS4*) of the primary *TAS* transcripts are the prerequisite for initiating ta-siRNA production. Through the RDR6-dependent pathway, the cleaved *TAS* transcripts are converted to double-stranded precursors which will be subject to ta-siRNA processing by DCL4. Finally, most of the ta-siRNAs are loaded into AGO1 silencing complexes to guide target cleavages (Peragine et al., 2004; Vazquez et al., 2004; Allen et al., 2005; Gasciolli et al., 2005; Xie et al., 2005; Yoshikawa et al., 2005; Rajagopalan et al., 2006). For the nat-siRNAs, genome-wide studies in both Arabidopsis and rice showed that they were originated from the overlapping regions of the natural antisense transcript (NAT) pairs through the DCL1-dependent pathway or through the Pol IV-, RDR2-, and DCL3-dependent pathway (Zhang et al., 2012). Moreover, in Borsani et al.’s study (2005), 21- and 24-nt nat-siRNAs were demonstrated to be produced from a *cis*-NAT pair through the RDR6- and DCL1/2-dependent pathway (Borsani et al., 2005). A major class of phasiRNAs was identified in the reproductive tissues of Gramineae species, such as rice (Johnson et al., 2009) and maize (*Zea mays*) (Zhai et al., 2015). Notably, in rice, the processing of 21-nt phasiRNAs was highly dependent on DCL4, while the processing of 24-nt ones required the activity of DCL3b (Song et al., 2012). Komiya and his colleagues (2014) reported that a portion of the DCL4-dependent, 21-nt phasiRNAs preferentially associated with MEL1 (the ortholog of Arabidopsis AGO5), and these 5′ C-started phasiRNAs originated from hundreds of lincRNA (long intergenic non-coding RNA) loci. In addition to the above mentioned sRNAs, some non-canonical sRNA species have been discovered, such as the AGO4-associated long siRNAs of 25-nt in length (Zilberman et al., 2003), the DCL3-dependent, AGO4-associated, 24-nt miRNAs called long miRNAs (Wu et al., 2010), the Pol IV- and DCL2/3/4-dependent, AGO2-associated double-strand break-induced sRNAs (Wei et al., 2012), the DCL-independent, AGO4-associated, 20- to 60-nt siRNAs (Ye et al., 2016), and the intron-derived, DCL2/3/4-dependent siRNAs (Chen et al., 2011). For a clear summarization, Fig. 1a provides a brief framework of the biogenesis and action pathways of the plant sRNAs. However, all of the recent discoveries just witnessed the emergence of the unexpectedly huge and complicated RNA world. It is still far from thorough understanding of the biogenesis and action pathways of the enormous sRNA population.

Fortunately, the valuable public resources have become increasingly available for the mechanistic studies on the plant sRNAs. Here, by taking the two model plants Arabidopsis and rice as an example, we provided a list of the currently available resources to the plant biologists, including the public databases, the bioinformatics softwares and the NGS datasets. Notably, most of the bioinformatics softwares listed here are online tools with user-friendly interface. By proposing a workflow for analyzing the biogenesis and action pathways of the plant sRNAs, we made a clear description for the specific applications of different sequencing datasets and bioinformatics toolkits at each analytical step. Finally, we anticipate that this workflow along with the list could advance the efficiency of data analysis and interpretation, thus facilitating the experimental design for the functional studies on the plant sRNAs. Below, we will introduce the public resources step by step according to the workflow shown in Fig. 1b.

### Genomic features and transcription

By using the BLAST tool provided by the plant genomic databases, such as TAIR (the Arabidopsis information resource) (Huala et al., 2001) for Arabidopsis and RGAP (rice genome annotation project) (Kawahara et al., 2013) or RAP-DB (the rice annotation project database) (Ohyanagi et al., 2006) for rice (Table 1), the genomic positions of the sRNA-coding loci could be obtained, facilitating the researchers to tell whether the sRNA loci are intergenic or intragenic. For mapping huge sRNA sequencing (sRNA-seq) datasets onto a plant genome, Bowtie should be selected as one of the powerful tools (Langmead et al., 2009). miRBase (the microRNA database) (Griffiths-Jones et al., 2006) and PLncDB (plant long non-coding RNA database) (Jin et al., 2013) are useful to check whether the sRNA is originated from a miRNA precursor or a lncRNA. Besides, ShortStack should be a useful tool to analyze the sRNA-seq data based on the available reference genomes (Axtell, 2013) (Table 2). It can output reports showing sRNA size distributions, repetitiveness, hairpin-association and phasing. One of its shortage is the requirement of bioinformatics experts for local installation and running.

**Table 1:** List of databases for plant small RNA research

Database	URL	Description	Reference
TAIR (the Arabidopsis information resource)	www.arabidopsis.org/	Genomic information database of Arabidopsis	(Huala et al., 2001)
RGAP (rice genome annotation project)	rice.plantbiology.msu.edu/	Genomic information database of rice	(Kawahara et al., 2013)
RAP-DB (the rice annotation project database)	rapdb.dna.affrc.go.jp/		(Ohyanagi et al., 2006)
Phytozome	phytozome.jgi.doe.gov/pz/portal.html	Genomic information of diverse plant species	(Goodstein et al., 2012)
miRBase (the microRNA database)	www.mirbase.org/	MicroRNA database of diverse organisms	(Griffiths-Jones et al., 2006)
PLncDB (plant long non-coding RNA database)	chualab.rockefeller.edu/gbrowse2/homepage.html	Long non-coding RNA database of Arabidopsis	(Jin et al., 2013)
PNRD (a plant non-coding RNA database)	(structuralbiology.cau.edu.cn/PNRD)	non-coding RNA database of diverse plant species	(Yi et al., 2015)
GEO (gene expression omnibus)	www.ncbi.nlm.nih.gov/geo/	Public repositories for next-generation sequence data	(Edgar et al., 2002)
SRA (sequence read archive)	www.ncbi.nlm.nih.gov/sra/		(Kodama et al., 2012)
DDBJ (DNA Data Bank of Japan) Sequence Read Archive	trace.ddbj.nig.ac.jp/dra/index_e.html		(Kaminuma et al., 2010)
Next-Gen Sequence Databases	mpss.danforthcenter.org/index.php	Public repositories for plant next-generation sequence data	(Nakano et al., 2006)
ASRP (Arabidopsis small RNA project)	asrp.danforthcenter.org/		(Gustafson et al., 2005)
CSRDB (cereal small RNAs database)	sundarlab.ucdavis.edu/smrnas/		(Johnson et al., 2007)
PlantNATsDB (plant natural antisense transcripts database)	bis.zju.edu.cn/pnatdb/	Database of plant natural antisense transcripts	(Chen et al., 2012)
mirEX (Arabidopsis pri-miRNA expression atlas)	www.combio.pl/mirex1/	Databases containing expression data of plant microRNA precursors	(Bielewicz et al., 2012)
PmiRKB (plant microRNA knowledge base)	bis.zju.edu.cn/pmirkb/		(Meng et al., 2011a)
AVT (AtGenExpress visualization tool)	jsp.weigelworld.org/expviz/expviz.jsp	Arabidopsis gene expression databases with user-friendly interface	(Kilian et al., 2007; Goda et al., 2008)
Arabidopsis eFP Browser	bar.utoronto.ca/efp_arabidopsis/cgi-bin/efpWeb.cgi		(Winter et al., 2007)
PceRBase (plant ceRNA database)	bis.zju.edu.cn/pcernadb/index.jsp	Database of plant competing endogenous RNAs	(Yuan et al., 2017)
Arabidopsis epigenome maps	neomorph.salk.edu/epigenome/epigenome.html	Arabidopsis epigenome maps	(Lister et al., 2008)
The SIGnAL Arabidopsis Methylome Mapping Tool	signal.salk.edu/cgi-bin/methylome		(Zhang et al., 2006)
UCSC Genome Browser on *Arabidopsis thaliana* (2004)	epigenomics.mcdb.ucla.edu/cgi-bin/hgTracks?clade = plant&org = A. + thaliana		(Zhang et al., 2006; Zhang et al., 2007)
UCSC Genome Browser on Arabidopsis thaliana (2009)	genomes.mcdb.ucla.edu/cgi-bin/hgTracks?db = araTha2		(Stroud et al., 2013)
Rice epigenome maps	(plantgenomics.biology.yale.edu)	Rice epigenome maps	(Li et al., 2008)
Plant Methylome DB	epigenome.genetics.uga.edu/PlantMethylome/	Database including epigenome data of 40 wild type plant species (presented by the Schmitz lab at the University of Georgia)	

Note: the currently invalid URL is noted by parentheses

**Table 2:** List of softwares for plant small RNA research

Software	URL	Description	Reference
PlantCARE (a plant *cis*-acting regulatory element database)	bioinformatics.psb.ugent.be/webtools/plantcare/html/	Plant gene promoter analysis	(Rombauts et al., 1999)
PLACE (a database of plant *cis*-acting regulatory DNA elements)	www.dna.affrc.go.jp/htdocs/PLACE/		(Higo et al., 1998)
JASPAR (an open-access database for eukaryotic transcription factor binding profiles)	jaspar.genereg.net		(Mathelier et al., 2016)
The MEME suite (containing motif-based sequence analysis tools)	meme-suite.org		(Bailey et al., 2015)
Bowtie	bowtie-bio.sourceforge.net/index.shtml	An ultrafast, memory-efficient short read aligner	(Langmead et al., 2009)
Bowtie 2	bowtie-bio.sourceforge.net/bowtie2/index.shtml	An ultrafast and memory-efficient tool for aligning relatively long sequencing reads to long reference sequences	(Langmead and Salzberg, 2012)
ShortStack	github.com/MikeAxtell/ShortStack/releases/	A Perl program for comprehensive annotation and quantification of small RNA genes	(Axtell, 2013)
NATpipe	www.bioinfolab.cn/NATpipe/NATpipe.zip	Natural antisense transcript prediction	(Yu et al., 2016a)
RNAfold webserver	rna.tbi.univie.ac.at/cgi-bin/RNAWebSuite/RNAfold.cgi	RNA secondary structure prediction	(Hofacker, 2003)
RNAshapes	bibiserv.cebitec.uni-bielefeld.de/download/tools/rnashapes.html		(Steffen et al., 2006)
miTRATA (microRNA truncation and tailing analysis)	wasabi.ddpsc.org/~apps/ta/index.php	3′ modification analysis of plant small RNAs	(Patel et al., 2016)
WebLogo	weblogo.threeplusone.com/	Search for the conserved sequence motifs	(Crooks et al., 2004)
psRNATarget (a plant small RNA target analysis server)	plantgrn.noble.org/psRNATarget/	Target prediction tools for plant small RNAs	(Dai and Zhao, 2011)
Small RNA Target Prediction	wasabi.ddpsc.org/~apps/tp/		(Jones-Rhoades and Bartel, 2004)
TAPIR (target prediction for plant microRNAs)	bioinformatics.psb.ugent.be/webtools/tapir/	Not only target prediction, also target mimic prediction for plant microRNAs	(Bonnet et al., 2010)
comPARE (PARE validated miRNA targets)	mpss.danforthcenter.org/tools/mirna_apps/comPARE.php	Degradome-seq data-based validation for plant microRNA—target pairs	(Kakrana et al., 2014)
sPARTA-Web (small RNA-PARE target analyzer)	mpss.danforthcenter.org/tools/mirna_apps/sparta.php	Degradome-seq data-based validation for plant small RNA—target pairs	
CleaveLand4	github.com/MikeAxtell/CleaveLand4/releases	A Perl program for degradome-seq data-based validation for plant small RNA—target pairs	(Addo-Quaye et al., 2009)
agriGO (a GO analysis toolkit for the agricultural community)	bioinfo.cau.edu.cn/agriGO/index.php	Functional analysis of target genes based on Gene Ontology annotations	(Du et al., 2010)
Cytoscape	www.cytoscape.org	Network data integration, analysis, and visualization	(Shannon et al., 2003)
Gephi	gephi.org		(Bastian et al., 2009)

Note: the currently invalid URL is noted by parentheses

Here, the workflow for analyzing the sRNA biogenesis and action pathways is proposed based on the scenario that the sRNAs are processed from their precursors transcribed from specific genomic loci (Fig. 1). If the sRNA precursor was experimentally cloned by using fine-scale methods such as RACE (rapid amplification of cDNA ends), the transcription boundary of the precursor-coding locus could be defined. In this case, the upstream region of user-defined length could be retrieved from the above mentioned genomic database, and be treated as the promoter region of this gene locus for *cis*-element analysis by using PlantCARE (a plant cis-acting regulatory element database) (Rombauts et al., 1999), PLACE (Plant cis-acting regulatory DNA elements database) (Higo et al., 1998), or the newly updated tools JASPAR (Mathelier et al., 2016) and the MEME suite (Bailey et al., 2015) (Table 2). The prediction results from these online tools might provide some valuable hints to infer the basic transcriptional features of this gene locus. For example, the coexistence of CAAT-box and TATA-box within the upstream region near to the transcription start site indicates the Pol II-drived transcription of the host gene (Lewin, 1990). Of course, we should acknowledge that the fine-scale cloning of the sRNA precursors is time consuming and laborious. The high-throughput solution is by analyzing the publicly available RNA sequencing (RNA-seq) data. Notably, distinct types of RNA-seq libraries were prepared with different purposes. For example, in a recent study, the poly(A)-tailed RNA-seq libraries were constructed for the detection of Pol II-dependent transcripts, while the rRNA-depleted total RNA-seq libraries were prepared for the identification of Pol IV-dependent transcripts (Li et al., 2015). After mapping such kind of RNA-seq data (Table 3) onto the plant genome by using a high-throughput alignment tool, Bowtie 2 (Langmead and Salzberg, 2012) for example, the transcription boundaries of the sRNA precursor-coding loci could be delineated. Also based on the mapping result, the RNA polymerase dependence could be partially determined for the loci. Moreover, some of the sRNA-seq datasets, such as those originated from the *nrpd1* mutant (a Pol IV mutant) (Table 3), could also be used to investigate the polymerase dependence of the sRNA-coding loci. Notably, compared to Bowtie, Bowtie 2 is particularly efficient for long read (up to hundreds of nucleotides in length) mapping. Thus, Bowtie 2 is recommended to be employed for RNA-seq data analysis, while Bowtie is more suitable for sRNA-seq read mapping as mentioned above.

**Table 3:** List of sequencing data for plant small RNA research

Data type	Species	Dataset ID		Description^a^	Reference
RNA-seq	Arabidopsis (Col-0)	GSE57215	GSM1377353	*dcl234* rep1	(Li et al., 2015)
			GSM1377354	*dcl234* rep2	
			GSM1377355	*dcl234* rep3	
			GSM1377356	*dcl234 nrpd1* rep1	
			GSM1377357	*dcl234 nrpd1* rep2	
			GSM1377358	*dcl234 nrpd1* rep3	
			GSM1377359	*dcl234* DSN	
			GSM1377360	*dcl234 nrpd1* DSN	
			GSM1377361	*dcl234 rdr2* DSN	
			GSM1377362	*dcl234* PolyA+ rep1	
			GSM1377363	*dcl234* PolyA+ rep2	
			GSM1377364	*dcl234 nrpd1* PolyA+ rep1	
			GSM1377365	*dcl234 nrpd1* PolyA+ rep2	
			GSM1377366	*dcl234* PolyA- rep1	
			GSM1377367	*dcl234* PolyA- rep2	
			GSM1377368	*dcl234 nrpd1* PolyA- rep1	
			GSM1377369	*dcl234 nrpd1* PolyA- rep2	
	Rice	GSE50778	GSM1229044	Nipponbare	(Wei et al., 2014)
			GSM1229045	Nipponbare *dcl3a* RNAi-1	
			GSM1229046	Nipponbare *dcl3a* RNAi-3	
DsRNA-seq	Arabidopsis	GSE23439	GSM575243	WT 1 × ribominus	(Zheng et al., 2010)
			GSM575244	WT 2 × ribominus	
			GSM575245	*rdr6*	
		GSE57215	GSM1377347	*dcl234* unopened flower buds rep1	(Li et al., 2015)
			GSM1377348	*dcl234* unopened flower buds rep2	
			GSM1377349	*dcl234* unopened flower buds rep3	
			GSM1377350	*dcl234 nrpd1* unopened flower buds rep1	
			GSM1377351	*dcl234 nrpd1* unopened flower buds rep2	
			GSM1377352	*dcl234 nrpd1* unopened flower buds rep3	
Degradome-seq	Arabidopsis (Col-0)	GSE77549	GSM2054358	WT 11-day-old seedlings	(Hou et al., 2016)
			GSM2054359	WT inflorescences	
			GSM2253889	WT inflorescences rep1	
			GSM2253892	WT inflorescences rep2	
			GSM2253890	*rdr6* inflorescences rep1	
			GSM2253893	*rdr6* inflorescences rep2	
			GSM2253891	*ago7* inflorescences rep1	
			GSM2253894	*ago7* inflorescences rep2	
		GSE52342	GSM1263708	WT inflorescences	(Creasey et al., 2014)
			GSM1263709	*ddm1-2* inflorescences	
			GSM1263710	*rdr6–15* inflorescences	
			GSM1263711	*ddm1–2 rdr6–15* inflorescences	
		GSE47121	GSM1145327	Flower buds rep1	(Willmann et al., 2014)
			GSM1145328	Flower buds rep2	
		GSE11007	GSM278333	Inflorescences, dT primed	(Addo-Quaye et al., 2008)
			GSM278334	Inflorescences, dT primed	
			GSM278335	Inflorescences, random primed	
			GSM278370	Seedlings, random primed	
		GSE11094	GSM280226	WT inflorescences	(German et al., 2008)
			GSM280227	*xrn4* inflorescences	
		GSE55151	GSM1330569	Young leaves	(Thatcher et al., 2015)
			GSM1330570	Mature leaves	
			GSM1330571	Early senescence leaves rep 1	
			GSM1330573	Early senescence leaves rep 2	
			GSM1330572	Late senescence leaves rep 1	
			GSM1330574	Late senescence leaves rep 2	
		GSE11070	GSM284751	WT Flowers rep1	(Gregory et al., 2008)
			GSM284752	*ein5-6* flowers rep1	
		GSE71913	GSM1847333	Unopened flower buds, abh1–1	(Yu et al., 2016b)
			GSM1847334	Unopened flower buds, abh1–8	
			GSM1939001	Leaves, treated with translation inhibitor CHX rep1	
			GSM1939002	Leaves, treated with translation inhibitor CHX rep2	
		GSE66224	GSM1617433	Immature inflorescences rep1	(Vandivier et al., 2015)
			GSM1617434	Immature inflorescences rep2	
	Rice	GSE42467	GSM1040649	Young panicles of ZH11 (japonica) at high temperature	
		GSE66610	GSM1626143	Nipponbare Leaves	(Baldrich et al., 2015)
			GSM1626145	Nipponbare Leaves	
		GSE62334	GSM1525457	Nipponbare leaves	
		GSE17398	GSM434596	Nipponbare seedlings	(Li et al., 2010)
		GSE19050	GSM476257	93-11 (indica) young inflorescences	(Zhou et al., 2010)
		GSE18251	GSM455938	Nipponbare seedlings	(Wu et al., 2009)
			GSM455939	Nipponbare inflorescences	
SRNA-seq	Arabidopsis (Col-0)	GSE57215	GSM1377370	WT rep1	(Li et al., 2015)
			GSM1377371	WT rep2	
			GSM1377372	*nrpd1* rep1	
			GSM1377373	*nrpd1* rep2	
			GSM1377374	*dcl3* rep1	
			GSM1377375	*dcl3* rep2	
			GSM1377376	*rdr2* rep1	
			GSM1377377	*rdr2* rep2	
			GSM1377378	*dcl234* rep1	
			GSM1377379	*dcl234* rep2	
			GSM1377380	*dcl234 nrpd1* rep1	
			GSM1377381	*dcl234 nrpd1* rep2	
		GSE23439	GSM575246	WT	(Zheng et al., 2010)
			GSM575247	*rdr6*	
		GSE14695	GSM366868	Whole aerials	(Fahlgren et al., 2009)
			GSM366869	Whole aerials *dcl1–7*	
			GSM366870	Whole aerials *dcl2–1dcl3-1dcl4–2*	
		GSE44622	GSM1087973	WT, flowers rep1	(Jeong et al., 2013)
			GSM1087974	WT, flowers rep2	
			GSM1087975	*dcl1–7*, flowers rep 1	
			GSM1087976	*dcl1–7*, flowers rep 2	
			GSM1087977	*dcl234*, flowers rep 1	
			GSM1087978	*dcl234*, flowers rep 2	
			GSM1087979	*rdr2*, flowers rep 1	
			GSM1087980	*rdr2*, flowers rep 2	
		GSE35562	GSM1178880	WT flowers, rep1	(Zhai et al., 2013)
			GSM1178881	WT flowers, rep2	
			GSM1178882	WT flowers, rep3	
			GSM1178883	*hen1–8* flowers, rep1	
			GSM1178884	*hen1–8* flowers, rep2	
			GSM1178885	*hen1–8* flowers, rep3	
		GSE26161	GSM642337	sRNAs cloned from total RNA	(Zhang et al., 2011)
			GSM642338	sRNAs cloned from AGO2	
		GSE28591	GSM707678	WT, flowers	(Wang et al., 2011)
			GSM707679	WT, leaves	
			GSM707680	WT, roots	
			GSM707681	WT, seedlings	
			GSM707682	AGO1-associated sRNAs, flowers	
			GSM707683	AGO1-associated sRNAs, leaves	
			GSM707684	AGO1-associated sRNAs, roots	
			GSM707685	AGO1-associated sRNAs, seedlings	
			GSM707686	AGO4-associated sRNAs, flowers	
			GSM707687	AGO4-associated sRNAs, leaves	
			GSM707688	AGO4-associated sRNAs, roots	
			GSM707689	AGO4-associated sRNAs, seedlings	
		GSE39885	GSM980695	sRNAs cloned from total RNA	(Zhu et al., 2011)
			GSM980697	sRNAs cloned from AGO10	
		GSE16545	GSM415783	sRNAs cloned from total RNA, flowers	(Havecker et al., 2010)
			GSM415784	sRNAs cloned from total RNA, flowers	
			GSM415785	sRNAs cloned from total RNA, flowers	
			GSM415791	sRNAs cloned from AGO9, flowers	
			GSM415792	sRNAs cloned from AGO9, flowers	
		GSE10036	GSM253622	sRNAs cloned from AGO1	(Mi et al., 2008)
			GSM253623	sRNAs cloned from AGO2	
			GSM253624	sRNAs cloned from AGO4	
			GSM253625	sRNAs cloned from AGO5	
		GSE12037	GSM304282	sRNAs cloned from total RNA (AGO2 mock)	(Montgomery et al., 2008)
			GSM304283	sRNAs cloned from AGO2	
			GSM304284	sRNAs cloned from total RNA (AGO7 mock)	
			GSM304285	sRNAs cloned from AGO7	
	Rice	GSE20748	GSM520640	Nipponbare WT seedlings	(Wu et al., 2010)
			GSM520639	Nipponbare *rdr2* seedlings	
			GSM520637	Nipponbare *dcl1* seedlings	
			GSM520638	Nipponbare *dcl3* seedlings	
		GSE26405	GSM648139	ZH11 (japonica) high temperature panicles	(Song et al., 2012b)
			GSM648140	ZH11 (japonica) *rdr6* high temperature panicles	
			GSM648141	ZH11 (japonica) low temperature panicles	
			GSM648142	ZH11 (japonica) *rdr6* low temperature panicles	
		GSE22763	GSM562942	93–11 (indica) WT seedlings	(Song et al., 2012a)
			GSM562944	93-11 (indica) *dcl4–1* seedlings	
			GSM562943	93–11 (indica) WT panicles	
			GSM562945	93–11 (indica) *dcl4–1* panicles	
		GSE35562	GSM913524	Dongjin hen1–3, leaves	(Zhai et al., 2013)
			GSM913525	Dongjin WT, leaves	
		GSE50778	GSM1229047	Nipponbare WT	(Wei et al., 2014)
			GSM1229048	Nipponbare *dcl3a*, RNAi-1	
			GSM1229049	Nipponbare *dcl3a*, RNAi-3	
		GSE32973	GSM816687	Nipponbare seedlings rep1	(Jeong et al., 2011)
			GSM816688	Nipponbare seedlings rep2	
			GSM816689	Nipponbare seedlings rep3	
			GSM816700	Nipponbare seedlings *dcl1* RNAi rep1–1	
			GSM816701	Nipponbare seedlings *dcl1* RNAi rep1–2	
			GSM816702	Nipponbare seedlings *dcl1* RNAi rep2–1	
			GSM816703	Nipponbare seedlings *dcl1* RNAi rep2–2	
			GSM816730	Nipponbare panicles rep1–1	
			GSM816731	Nipponbare panicles rep1–2	
			GSM816732	Nipponbare panicles rep2	
			GSM816745	Nipponbare panicles *dcl1* RNAi rep1–1	
			GSM816746	Nipponbare panicles *dcl1* RNAi rep1–2	
			GSM816747	Nipponbare panicles *dcl1* RNAi rep2–1	
			GSM816748	Nipponbare panicles *dcl1* RNAi rep2–2	
		GSE20748	GSM520640	Nipponbare seedlings, sRNAs cloned from total RNA	(Wu et al., 2010)
			GSM520634	Nipponbare seedlings, sRNAs cloned from AGO4a	
			GSM520635	Nipponbare seedlings, sRNAs cloned from AGO4b	
			GSM520636	Nipponbare seedlings, sRNAs cloned from AGO16	
		GSE18250	GSM455962	Nipponbare seedlings, sRNAs cloned from AGO1a	(Wu et al., 2009)
			GSM455963	Nipponbare seedlings, sRNAs cloned from AGO1b	
			GSM455964	Nipponbare seedlings, sRNAs cloned from AGO1c	
		PRJNA273330	SRX847816	Nippbare sRNAs cloned from AGO1a Rep1	
			SRX847817	Nippbare sRNAs cloned from AGO1a Rep2	
			SRX847818	Nippbare sRNAs cloned from AGO1b Rep1	
			SRX847819	Nippbare sRNAs cloned from AGO1b Rep2	
			SRX847820	Nippbare sRNAs cloned from AGO18 Rep1	
			SRX847821	Nippbare sRNAs cloned from AGO18 Rep2	
		DRP000161	DRX000196	sRNA-IP in WT (Nipponbare)	(Komiya et al., 2014)
			DRX000197	sRNA-IP in mel1 (Nipponbare)	
			DRX000198	Total sRNA in WT (Nipponbare)	
			DRX000199	Total sRNA in mel1 (Nipponbare)	

^a^Please see detailed descriptions of the datasets in the related references

### Precursor formation and processing

As introduced above, there are two major forms of sRNA precursors that could be processed by DCLs, i.e. the long double-stranded RNA (dsRNA) precursors and the single-stranded RNAs with short internal double-stranded regions (Fig. 1). The former ones could be synthesized either through the RDR-dependent (such as the precursors of the hc-siRNAs or the ta-siRNAs) pathway or through the RDR-independent (such as the NATs) pathway. However, the later ones are unexceptionally generated through the RDR-independent pathways (such as the pri-miRNAs and the sirtrons). Thus, distinct bioinformatics toolkits should be selected to identify the sRNA precursors belonging to the two different types, respectively.

PlantNATsDB (plant natural antisense transcripts database) (Chen et al., 2012) provides the users with the genome-wide prediction results of both *cis*- and *trans*-NAT pairs of 70 plant species. Gene locus ID could be used as a query to see the possibility of this gene to form NAT pairs with other genes. Optionally, “batch download” could be selected to obtain the complete list of the predicted NAT pairs of a plant species. In the other way, the researchers could perform large-scale NAT prediction by using the program NATpipe (Yu et al., 2016a). The genome-independent feature of this software allows users to carry out NAT prediction solely based on the RNA-seq data. If the genomic information is available, then the predicted NATs could be classified into *cis* and *trans* ones. Additionally, if the sRNA-seq data is available, phase-distributed nat-siRNAs could be identified from the predicted NATs by using NATpipe.

RNAfold (Hofacker, 2003) and RNAshapes (Steffen et al., 2006) are both easy-to-use tools for local RNA secondary structure predictions. RNAfold is a web server allowing a query sequence of up to 10,000 nt in length, but its graphic outputs are difficult to be modified according to the users’ requirements. RNAshapes is a locally installed program with a strict length limitation (up to ~400 nt based our experience) of an input sequence. However, the outputs of RNAshapes could be graphically edited.

Recently, NGS-based, transcriptome-wide strategies have been developed to probe the RNA secondary structures, such as dsRNA sequencing (dsRNA-seq) (Kwok et al., 2015). The dsRNA-seq library is prepared by treating the total RNAs with the ribonuclease specific for the single-stranded RNAs, thus enabling researchers to detect the annealed region within an RNA sequence, or between two transcripts. Currently, the plant dsRNA-seq data is only available for Arabidopsis. These dsRNA-seq datasets were prepared not only from the wild type (WT) plants, but also from the *nrpd1* and *rdr6* mutants (Table 3). Thus, the Pol IV and RDR6 dependence of the dsRNA precursors could be interrogated by using these datasets. In addition to the dsRNA-seq data, sRNA-seq data of *nrpd1*, *rdr2* and *rdr6* should also be valuable to investigate the biogenesis pathways of the sRNA precursors (Table 3).

DCLs have been demonstrated to be widely implicated in the processing of diverse sRNA precursors in plants (Chen, 2009). Thus, by comparing to the sequencing data of WT, the public sRNA-seq data of the *dcl* mutants could be used to investigate the specific DCL-mediated sRNA processing pathways.

### sRNA action modes and network construction

According to the current understanding, target cleavages and chromatin modifications are the two major action modes of the plant sRNAs. And, these two distinct regulatory pathways are largely predetermined by the association of the sRNAs with specific AGO complexes (Fang and Qi, 2016). Thus, AGO enrichment analysis is necessary for functional studies on the plant sRNAs. To date, sequencing data of the AGO-associated sRNA populations has been reported by several research groups (Table 3). In Arabidopsis, AGO1-, AGO2-, AGO4-, AGO5-, AGO7-, AGO9- and AGO10-associated sRNA sequencing datasets are available (Mi et al., 2008; Montgomery et al., 2008; Havecker et al., 2010; Wang et al., 2011; Zhu et al., 2011). And in rice, AGO1-, AGO4-, MEL1-, AGO16- and AGO18-associated sRNA sequencing datasets have been published (Wu et al., 2009; Wu et al., 2010; Komiya et al., 2014). By comparing the level of a sRNA in a specific AGO complex to that in the total RNA extract, whether this sRNA is enriched in the AGO complex could be determined. The result could facilitate the researchers to deduce the action mode of this sRNA.

A large portion of the miRNAs and some of the siRNAs such as the ta-siRNAs are incorporated into AGO1. These AGO1-associated sRNAs can recognize the highly complementary regions on the target transcripts, and inhibit gene expression through target cleavages. The high complementarity between the sRNA and its target forms an essential basis for the development of the target prediction tools. For plants, there are several user-friendly online tools for target prediction (Table 2), such as psRNATarget (a plant small RNA target analysis server) (Dai and Zhao, 2011), Small RNA Target Prediction (Jones-Rhoades and Bartel, 2004), and TAPIR (target prediction for plant microRNAs) (Bonnet et al., 2010). Compared to TAPIR, the former two tools are more flexible for different users’ purposes. By using psRNATarget or Small RNA Target Prediction, the users can select one of the cDNA libraries provided by the tools, or can upload their own cDNA sequences for target prediction. However, TAPIR does not provide the pre-stored cDNA libraries for the users. Additionally, much more parameters are adjustable before performing analysis by using the former two tools. Thus, psRNATarget and Small RNA Target Prediction should be the efficient and easy-to-use tools for plant sRNA target prediction.

The 3′ cleavage remnants from the target transcripts are relatively stable in vivo, and could be detected by sequencing. This kind of high-throughput sequencing technology was called GMUCT (global mapping of uncapped and cleaved transcripts) (Willmann et al., 2014) or PARE (parallel analysis of RNA ends) (German et al., 2008; German et al., 2009), which is collectively referred to as degradome sequencing (degradome-seq) here. As listed in Table 3, there are many degradome-seq datasets available to perform large-scale validation of the predicted sRNA targets. For analyzing the degradome-seq data, comPARE (PARE validated miRNA targets) and sPARTA-Web (small RNA-PARE target analyzer) (Kakrana et al., 2014) might be the easy-to-use online tools for the wet-lab researchers (Table 2). The difference between comPARE and sPARTA-Web is that the former was designed specifically for the miRNA target validation whereas the latter was developed for all of the sRNA target candidates. CleaveLand4 (Addo-Quaye et al., 2009) is also suitable for degradome-seq data-based validation of the sRNA targets. However, it is a Perl program, which requires extensive support from bioinformatics experts for local installation and running. Besides, our previously proposed workflow could also be referenced for degradome-seq data-based sRNA target validation (Meng et al., 2011b).

The AGO4-associated sRNAs, such as the hc-siRNAs, repress gene expression through chromatin modifications (Chen, 2009; Fang and Qi, 2016). By using BLAST or Bowtie, the genomic regions highly complementary to the AGO4-associated sRNAs could be identified with a genome-wide scale. Then, it will be interesting to investigate the chromatin status surrounding the complementary sites. Several epigenome databases are available for Arabidopsis, such as Arabidopsis epigenome maps (Lister et al., 2008), the SIGnAL Arabidopsis Methylome Mapping Tool (Zhang et al., 2006), and the Arabidopsis epigenome data displayed in the UCSC Genome Browser (Zhang et al., 2006; Zhang et al., 2007; Stroud et al., 2013). In some databases, in addition to the WT data, the epigenomes of diverse mutants are also available, which might be valuable to inspect the sRNA-guided chromatin modification pathways in detail. Although the rice epigenome data was reported nearly ten years ago (Li et al., 2008), and the database was established at that time, the web link is no long active. Fortunately, Plant Methylome DB provides researchers with the WT epigenomes of 40 species including Arabidopsis and rice.

“Target mimicry” was reported as a novel pathway for the regulation of the miRNA activities by the target mimics (Franco-Zorrilla et al., 2007). Although the online server TAPIR is not superior in sRNA target prediction, it provides a unique functional module for target mimic prediction (Bonnet et al., 2010). Besides, the recently established PceRBase (plant ceRNA database) stores the lists of the competing endogenous RNAs (similar to the target mimics) of 26 plant species for the users (Yuan et al., 2017).

Finally, researchers could construct a sRNA-centered regulatory network involving sRNA targets and target mimics by using Cytoscape (Shannon et al., 2003) or Gephi (Bastian et al., 2009). The expression levels of the sRNA precursors, the sRNA target genes and the target mimics could be partially uncovered by visiting PmiRKB (plant microRNA knowledge base) (Meng et al., 2011a), mirEX (Arabidopsis pri-miRNA expression atlas) (Bielewicz et al., 2012), AVT (AtGenExpress visualization tool) (Kilian et al., 2007; Goda et al., 2008), and Arabidopsis eFP Browser (Winter et al., 2007). The biological functions of the sRNA target genes could be analyzed by using agriGO (Du et al., 2010).

## Conclusions

In the present work, we proposed a general workflow for deciphering the biogenesis and action pathways of the plant sRNAs by using a series of publicly available resources. Most of the recently reported sRNA-seq and dsRNA-seq datasets of Arabidopsis and rice were summarized in Table 3, emphasizing their importance for elucidating the RDR- and DCL-dependent biogenesis pathways of the plant endogenous sRNAs. However, we should acknowledged that several useful toolkits have not been included in the list of softwares for plant small RNA research. For example, the UEA sRNA workbench, that is downloadable for local installation, provides an user-friendly platform for sRNA-seq data processing (Stocks et al., 2012). It contains several useful tools, such as “adaptor remover” and “Filter” for sRNA-seq data pre-treatment, “miRCat” and “hairpin annotation” for miRNA prediction, and the ta-siRNA prediction tool. Besides, as noticed in Tables 1 and 2, several valuable databases and bioinformatics tools, including Rice epigenome maps and PNRD, are currently terminated for unknown reason. We hope that these useful resources could be activated again for the plant biologists. Summarily, more research efforts, from both the bioinformaticians and the experimental practitioners, are anticipated to devote to the plant sRNA research.

## Abbreviations

AGO: Argonaute
AVT: AtGenExpress visualization tool
DCL: Dicer-like
degradome-seq: degradome sequencing
dsRNA: double-stranded RNA
dsRNA-seq: double-stranded RNA sequencing
GMUCT: Global mapping of uncapped and cleaved transcripts
hc-siRNA: heterochromatic small interfering RNA
lincRNA: long intergenic non-coding RNA
lncRNA: long non-coding RNA
miRNA: microRNA
NAT: Natural antisense transcript
nat-siRNA: natural antisense transcript-derived small interfering RNA
ncRNA: non-coding RNA
NGS: Next-generation sequencing
PARE: Parallel analysis of RNA ends
PceRBase: Plant ceRNA database
phasiRNA: phased small interfering RNA
PlantCARE: a plant cis-acting regulatory element database
PlantNATsDB: Plant natural antisense transcripts database
PLncDB: Plant long non-coding RNA database
PmiRKB: Plant microRNA knowledge base
Pol II: RNA polymerase II
poly(A): polyadenylation
pri-miRNA: primary transcript of microRNA
psRNATarget: a plant small RNA target analysis server
RACE: rapid amplification of cDNA ends
RAP-DB: the rice annotation project database
RDR: RNA-dependent RNA polymerase
RGAP: Rice genome annotation project
RNA-seq: RNA sequencing
rRNA: ribosomal RNA
siRNA: Small interfering RNA
sRNA: Small RNA
sRNA-seq: sRNA sequencing
TAIR: the Arabidopsis information resource
TAPIR: Target prediction for plant microRNAs
ta-siRNA: trans-acting small interfering RNA
WT: Wild type
